# Editorial Comment: Efficiency and satisfaction with telephone consultation of follow-up patients in neuro-urology: Experience of the COVID-19 pandemic

**DOI:** 10.1590/S1677-5538.IBJU.2022.01.04

**Published:** 2021-12-21

**Authors:** Marcio Augusto Averbeck

**Affiliations:** 1 Hospital Moinhos de Vento Unidade de Videourodinâmica Porto Alegre RS Brasil Chefe de Neuro-Urologia, Unidade de Videourodinâmica, Hospital Moinhos de Vento. Porto Alegre, RS, Brasil

Camille Chesnel 1, Claire Hentzen 1, Frédérique Le Breton 1, Nicolas Turmel 1, Eliane Tan 1, Rebecca Haddad 1 , Gérard Amarenco 1

Neurourol Urodyn. 2021 Mar;40(3):929-937.

## COMMENT

The new acute respiratory syndrome coronavirus type 2 (SARS-CoV-2) and the disease it causes, coronavirus disease 19 (COVID-19), were initially described in Wuhan, China, on December 20191. SARSCoV-2 spread worldwide and the World Health Organization (WHO) announced a pandemic on March 11th 2020. Over the last year, COVID-19 has dramatically changed standard urology practice in all continents. Due to the inherent risk of contagion and to the potential depletion of health services, medical care has focused on new models to avoid face-to-face contact between the clinician and the patient.

Chesnel et al. aimed to assess the efficiency and satisfaction of a telephone consultation in neuro‐urology during the pandemic. The study was conducted in a neuro‐urology department of a French university hospital between March 16th and June 1st 2020. Scheduled medical visits were converted into telephone consultation. For each teleconsultation, the physician assessed the efficiency and the patient‐rated global satisfaction of the teleconsultation. Physicians conducted the teleconsultations by telephone, without the use of video tools. For each teleconsultation, the physician reported the duration of the consultation and the number of tries to join the patient. The physician rated on a numerical scale of 0–10, the efficiency of the consultation, that is, the ability to understand, to analyze the patient’s symptoms, to rule out an urgent situation, to propose a therapeutic adaptation, and to plan a later appointment.

Three hundred twenty‐eight out of 358 patients (91.6%) evaluated the telephone consultation and were included in the study. The mean efficiency of the telephone consultation was 9.3/10 (±1.5). The mean global satisfaction was 9.0/10 (±1.3). The majority of the patients (52.4%) would prefer a physical consultation. 90.2% might convert some clinic visits to teleconsultations in the future. No agreement was found between the patient and the physician when they were asked if the teleconsultation replaced the physical consultation initially scheduled (weight kappa=0.02; 95% confidence interval=[−0.06 to 0.11]). Cognitive impairment, difficulty to obtain relevant information, and lack of physical examination were unfavorable to the efficiency of the teleconsultation. Cognitive impairment, embarrassing nature of the teleconsultation, and preference for a physical consultation were unfavorable to patient’s satisfaction. Telemedicine in neuro-urology was associated with a high satisfaction of the patients and was described as efficient by the physicians. Despite this, the majority of the patients reported a preference for physical consultation.

During the long-lasting COVID-19 pandemic, we have learned that not all clinical situations are appropriate for video consultations. Both the value and the ultimate contribution of telehealth in functional urology have yet to be further assessed, but this approach will be of age and may provide an effective method for follow-up in neuro-urology, especially when physical examination or other testing methodologies are not promptly required.
